# Socioeconomic inequalities in maternal health care utilization in Ghana

**DOI:** 10.1186/s12939-019-1043-x

**Published:** 2019-09-05

**Authors:** Jacob Novignon, Bernice Ofori, Kwasi Gyabaa Tabiri, Mohammad Habibullah Pulok

**Affiliations:** 1 Department of Economics, Kwame Nkrumah University of Science and Technology, Kumasi, Ghana; 2 School of Health Administration, Dalhousie University, Halifax, NS Canada; 3 Geriatric Medicine Research, Nova Scotia Health Authority, Halifax, NS Canada

**Keywords:** Maternal health, Inequality, Universal health coverage, Ghana

## Abstract

**Background:**

Improving maternal and child health remains a public health priority in Ghana. Despite efforts made towards universal coverage, there are still challenges with access to and utilization of maternal health care. This study examined socioeconomic inequalities in maternal health care utilization related to pregnancy and identified factors that account for these inequalities.

**Methods:**

We used data from three rounds of the Ghana Demographic and Health Surveys (2003, 2008 and 2014). Two health care utilization measures were used; (i) four or more antenatal care (ANC) visits and (ii) delivery by trained attendants (DTA). We first constructed the concentration curve (CC) and estimated concentration indices (CI) to examine the trend in inequality. Secondly, the CI was decomposed to estimate the contribution of various factors to inequality in these outcomes.

**Results:**

The CCs show that utilization of at least four ANC visits and DTA were concentrated among women from wealthier households. However, the trends show the levels of inequality decreased in 2014. The CI of at least four ANC visits was 0.30 in 2003 and 0.18 in 2014. Similarly, the CIs for DTA was 0.60 in 2003 and 0.42 in 2014. The decomposition results show that access to National Health Insurance Scheme (NHIS) and women’s education levels were the most important contributors to the reduction in inequality in maternal health care utilization.

**Conclusions:**

The findings highlight the importance of the NHIS and formal education in bridging the socioeconomic gap in maternal health care utilization.

## Background

Improving maternal health remains an important global health priority. The Sustainable Development Goals (SDGs) that replaced the Millennium Development Goals (MDGs) include maternal health and health care as overarching goals. Moreover, the SDGs emphasize that no one should be left behind in the achievement of these goals [20]. This requires sufficient and sustainable efforts to remove various barriers to health care access and utilization, particularly in developing countries.

There have been several efforts in recent times to improve maternal health through the removal of barriers that limit access and utilization of health services. Prominent among these is the call for universal health coverage (UHC) which is defined by the World Health Organization (WHO) as a state where people and communities can use health care services they need without any financial hardship [23]. While promoting UHC in developed countries is a novel approach, it requires significant efforts to remove various barriers that limit access and utilization of health care, especially for the poor and vulnerable. Evidence suggests that socioeconomic inequalities are high in developing countries where health systems are largely under developed [5, 12, 14]. In most cases, the poor are disproportionately affected by these inequalities.

The literature on socioeconomic inequality in maternal health care utilization in low-middle income countries suggest that wealth-related inequality in maternal care has increased in some developing countries [2, 17]. There is also growing evidence that show inequalities are particularly high in many countries in Africa compared to other regions [1, 9]. For instance, Obiyan and Kumar [14] showed that in Nigeria, there were significant socioeconomic differences in the utilization of antenatal care (ANC) as well as medical care assistance at delivery between 1990 and 2008.

In Ghana, while maternal health has generally improved over the years, there still remain significant room for improvement. Available estimates suggest that maternal mortality rate was 319 per 100,000 live births in 2015. This is relatively lower than SSA average (547 per 100,000 live births) but above the average for Lower Middle Income countries (260) and global average of 216 [24]. Averting this situation requires local efforts directed towards improving access and utilization of maternal health care in Ghana. This includes removing financial barriers and resultant inequalities in health care utilization. An important policy effort in this regard is the Ghana National Health Insurance Scheme (NHIS). The scheme is touted as the largest and most important health financing reform in the history of the country. The primary objective of the scheme was, among others, to remove financial barriers to health care access across the country. The scheme also has, as its core mandate, to reduce socioeconomic inequalities in health care utilization by providing financial risk protection to the poor and the vulnerable. This objective is pursued by the various exemption policies administered by the scheme. For instance, the poor, pregnant women, and the aged are among those exempted from paying premium [4]. The ultimate goal is to improve health by reducing socioeconomic inequalities. In particular, the exemption of pregnant women from paying premium was notable with the objective of encouraging antenatal care service and facility delivery among women. This was expected to help mitigate maternal mortality from pregnancy and delivery related complications. The policy also sought to encourage poor and vulnerable women to seek pregnancy related health care.

Few studies have analyzed socioeconomic inequalities in various measures of maternal health utilization in Ghana. Most recently, Fenny et al. [8] estimated trends and determinants of inequality in ANC timing, number of ANC visits, and place of delivery. They found pro-rich inequality in all three measures and attributed the reduction in inequality to user fee removal for maternal health services. In contrast, Asamoah and Agardh [3] found that wealth-related inequality in antenatal care (ANC) service utilization increased between 2003 and 2008. It should be noted that while both Fenny et al. [8] and Asamoah and Agardh [3] used the same demographic and health survey (DHS) dataset, the methods of analysis differ. Even though this disparity may explain contrasting findings, there is need for further evidence.

Therefore, this study aims to examine socioeconomic inequalities in ANC utilization and delivery by trained attendants (DTA). Specifically, our research questions were two-fold; (i) what are the trends and patterns in socioeconomic related inequalities in ANC utilization and DTA? (ii) What factors contribute to socioeconomic related inequalities in ANC utilization and DTA? Our study differs from the two studies discussed above in two distinct ways. Our methods differ from Asamoah and Agardh [3], as we decomposed our inequality estimates to identify the main contributors of inequality. Our measures of health care utilization also differ from that of Fenny et al. [8]. While Fenny et al. [8] used place of delivery as a measure of maternal health care utilization, we used delivery attended by a skilled birth attendant.

## Methods

### Data

The study employed secondary data from three rounds of the Ghana Demographic and Health Survey (GDHS) conducted between 2003 and 2014. In Ghana, the survey was conducted by the Ghana Statistical Service (GSS) in collaboration with the National Public Health and Reference Laboratory (NPHRL) and the Ghana Health Service (GHS).

The survey collects comprehensive information on health care utilization. It also collects information on household asset and ownership. This serves as an important source to assess economic status of the household. We used the wealth index as the measure of socioeconomic status to rank women from the lowest to highest in the inequality analysis. In this study, we used three rounds of the GDHS survey (2003, 2008 and 2014). The Ghana Demographic and Health Survey (GDHS) followed a two-stage sample design. The first stage involved selecting sample points (clusters) consisting of enumeration areas (EAs). The second stage involved the systematic sampling of households. The households included in the survey were randomly selected from each cluster to constitute the total sample size of households. In deriving the data, focus was on women aged 15 to 49 who were permanent residents of the household or visitors who had stayed in the household being interviewed the night preceding the Survey (GSS, 2015). A total of 5691 eligible women participated in the 2003 survey whilst 4916 women as well as 9396 women were interviewed in the 2008 and 2014 survey respectively. The survey year 2003 represents the period before the introduction of the NHIS in 2004 while 2008 and 2014 represent two periods after the implementation of the scheme.

### Analytical approach

The analytical approach for this study was in three stages. The first stage used concentration curves (CCs) to examine the trend and pattern of socioeconomic inequalities in health care utilization (measured by antenatal care and delivery by trained attendants). In the second stage, concentration indices (CIs) were computed for each outcome variable across the years. The final stage decomposed the concentration indices to understand the contribution of various factors to inequality. The methods are discussed in detail as follows.

### Concentration curves and concentration indices

To examine the trend in inequalities in health care utilization, we constructed CC for each of the health care utilization measures. The CC gives a graphical view of the pattern and extent of inequalities in ANC utilization and DTA. A CC is a plot of the cumulative percentage of the outcome variable on the y-axis against the cumulative percentage of the population ranked by household socioeconomic status (starting from the poorest) on the x-axis [15]. The 45^0^ line or the diagonal in the CC graph represents equality in healthcare utilization. If the CC lies above the diagonal, outcome variable is concentrated among poorer people. When it is concentrated among richer people, the CC lies below the line of equality. There is no inequality when the CC lies on the 45° line. The extent of inequality is shown by how far the CC lies away from the line of equality (45° line). The further the CC is from the line of equality, the greater the extent of inequality [15]. The CIs were also estimated to determine the degree and nature of inequalities in ANC and DTA.

The CI is defined as “two times the area between the concentration curve and the line of equality” ([15], p. 95). The CI was calculated using the following formulae.

1 $$ \mathrm{CI}=\frac{2}{\mu }c\; ov\;\left({y}_i{r}_i\right) $$

Where *y* is a set of health utilization variables, *r*_*i*_ is fractional rank of individual in the wealth score distribution, *cov* is covariance and *μ* represents the mean of the healthcare variable. The CI can either be positive or negative. The sign of the CI explains the relationship that exists between the healthcare variable and position in the wealth score distribution. If the CI is zero, it means that there is no inequality in the distribution of healthcare use by wealth and hence the CC will coincide with the line of equality. A negative value of the CI is obtained if the healthcare variable is disproportionately concentrated among the poorest whilst a positive value of CI suggest inequality concentrated among the richest. The value of the CI ranges between − 1 and + 1 (i.e., − 1 ≤ CI ≤1) and the CI gives information about the strength of the relationship and the extent of variability in the dependent variables. The closer the absolute value of the CI to one, the greater the level of inequality.

### Decomposition analysis

The decomposition of the CI was performed to estimate the individual contribution of explanatory variables to inequalities in the outcome variables. The contribution of every individual characteristic is defined as the product of how sensitive that characteristic is to health and the extent of inequality in that factor [21].

Decomposition of the healthcare inequality relies on the assumption that the healthcare is a linear function of the outcome variables. This is important because in decomposition analysis the concentration indices are calculated using the predictions from a linear regression model [21].

The starting point was to express a linear function of the outcome variables in relation to the NHIS variable as well as other demographic and socioeconomic control variables. This is given as:

2 $$ y=\mathrm{a}+{\sum}_k{\beta}_k{x}_k+\upvarepsilon $$

Where x represents the vector of explanatory variables, including NHIS status. Following Wagstaff, Doorslaer, & Watanabe [21], the standard concentration index (CI) for outcome variable *y* can be written as

3 $$ \mathrm{CI}\left(\mathrm{y}\right)={\sum}_k\left({\beta}_k{\overline{x}}_k/\upmu \right)\;{c}_k+\mathrm{G}{C}_{\varepsilon }/\upmu $$

Where CI(*y*) is the standard concentration index, ${\overline{x}}_k$ is the mean of *x*_*k*_, *c*_*k*_ is the CI for *x*_*k*_, *μ* is the mean of y, G *C*_*ε*_ is the generalized CI for the error term (ε). From equation (3), two important grouping can be made; (i) the first term on the right-hand side of the equation expresses a weighted sum of the CI of *k* regressions, where the weight ${\overline{x}}_k$ is the elasticity of *y* with respect to *x*_*k*_ (*η*_*k*_ = ${\beta}_k{\overline{x}}_k$ / *μ)*. (ii) the second term on the right-hand side is the residual element which expresses the portion of inequality that cannot be explained by the contributing variables. Statistical significance of the CIs as well as the decomposition analysis was calculated using the bootstrapping technique with robust standard errors [7].

### Description of variables

In this study, we focused on pregnancy-related maternal health care utilization indicators. Specifically, we used, at least four ANC visits, and delivery by skilled attendants. Outcomes were measured as dummy variables that take the value of one if a woman had utilized the service and 0 otherwise.

In terms of socioeconomic indicators, the GDHS collects household asset information that is used to compute a wealth index. This wealth index has been shown to be strongly correlated with the economic and social status of the household [18]. Other variables included community, household and individual characteristics. The GDHS collects demographic and socioeconomic variables such as education, place of residence, age, gender, health facility in community, health insurance coverage, region of residence, sanitation, family size, among others.

## Results

### Descriptive statistics

We present summary statistics for all variables included in the study in Table 1. The proportion of women who attended minimum four antenatal visits increased from 70.6% in 2003 to 78.7% in 2008 and further to 86.5% in 2014. In addition, the proportion of delivery by skilled attendants increased from 43.9% in 2003, to 67.8% in 2008, and then to 72.8% 2014. About 70.6% of women included in the survey in 2003 were from rural areas. The percentage decreased gradually over the period from 64.5% in 2008 to 58.6% in 2014.The percentage of women from poor households was 32.1% in 2003 and this decreased to 29.6% in 2008. However, there was a marginal increase (30.7%) in this proportion in 2014. The proportion of women from households in the richest quintile however fell between 2003 and 2008 from 13.5% to 12.8% but saw a slight increase to 12.9% in 2014.

**Table 1:** Descriptive statistics

Variable	2003	2008	2014
Number of ANC visits			
At least four visits	0.706	0.787	0.865
At least one visit	0.921	0.961	0.969
Delivery by trained attendants	0.439	0.678	0.728
Rural	0.706	0.645	0.586
*Wealth Status*			
Poor	0.321	0.296	0.307
Poorer	0.213	0.220	0.215
Middle	0.181	0.175	0.189
Richer	0.150	0.182	0.160
Richest	0.135	0.128	0.129
*Education*			
No Education	0.458	0.361	0.330
Primary	0.208	0.237	0.202
Secondary	0.323	0.381	0.428
Higher	0.011	0.022	0.039

Source: Authors’ computation from GDHS data

The proportion of women with no education decreased over the period from 45.8% in 2003 to 33% in 2014. In addition, women who had achieved a level of education higher than secondary education increased from 1.1% in 2003 to 2.2% in 2008, and then to 3.9% in 2014.

### Trends and patterns of socioeconomic inequality in ANC and DTA

**Fig. 1:**
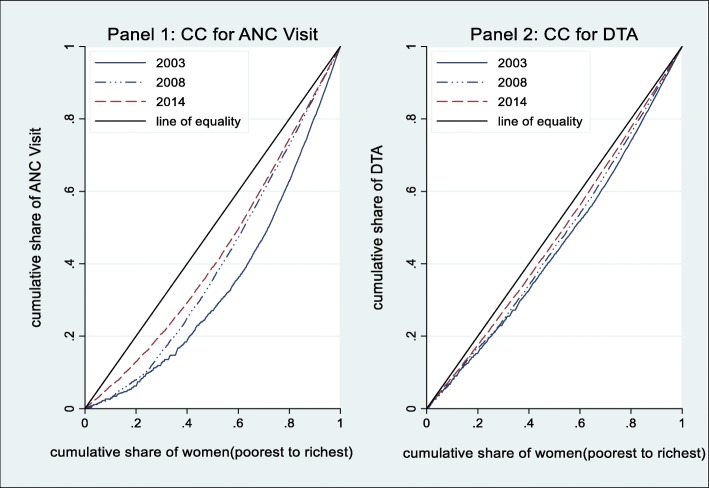
Concentration curves for ANC and DTA in Ghana. Panel 1: CC for ANC Visit. Panel 2: CC for DTA

Figure 1 shows CCs of the outcome variables over time. For each panel, the CC for the outcome variable was constructed for the years 2003, 2008 and 2014. Panel 1 shows that socioeconomic-related inequality in at least four ANC visit was in favor of women from wealthy households. The level of inequality declined across the years as the CC of at least four ANC visits became closer to the line of inequality between 2003 and 2014.

Panel 2 shows CCs of DTA. Again, the curves show evidence of socioeconomic inequality in favor of women from wealthy households. The nature of the CCs also suggest that the levels of inequality was higher for DTA compared to ANC visits. The curves also show that over the years there was a decline in the level of inequality. The curve for 2014 lies closest to the line of equality while the curve for 2003 lies furthest from the line of equality.

While the CCs provide a clear pictorial view of the levels and nature of inequality in the outcome variables, it does not provide the magnitude of inequality. To address this, we compute and report the concentration indices in Table 2. As mentioned earlier, the positive sign of the CI suggests that socioeconomic inequality favored the privileged (or was concentrated against the poor). The closer the index to one, the larger the level of inequality. Results in Table 2 suggest that inequality in at least four ANC visits and DTA was in favor of the rich or privileged. This confirms earlier findings from the concentration curves.

**Table 2:** Concentration indices for ANC and DTA in Ghana, 2003–2014

Outcome variables	2003	2008	2014
ANC required	0.303***(0.025)	0.257***(0.023)	0.177***(0.024)
Delivery assisted by trained attendants (DTA)	0.598***(0.026)	0.560***(0.025)	0.424***(0.024)

Robust standard errors are reported in parenthesis. ***p < 0.01

We also observed that for all the pregnancy-related maternal health care utilization measures, inequality declined consistently over the years. For ANC visit (measured as at least four visits), the CI was 0.30 in 2003, 0.26 and 0.18 in 2008 and 2014, respectively. For DTA, the CI was initially at 0.60 in 2003, 0.56 and 0.42 in 2008 and 2014 respectively. Further, the magnitude of inequality was higher for DTA than at least four ANC visits. All the estimates are statistically significant at 1%.

### Decomposinginequality in ANC and delivery assisted by trained attendants

To understand the factors that contribute to inequality, we decomposed the estimates reported in Table 2. These results are reported in Tables 3 and 4.The tables present results for 2008 and 2014.^1^ For each year, the elasticity, the CI and absolute contribution of the explanatory variables are reported.

**Table 3:** Contributing factors of inequality in at least for 4 ANC visits in Ghana, 2008–2014

Variables	2008			2014		
	Elasticity	Concentration Index	Absolute contribution	Elasticity	Concentration Index	Absolute contribution
Place of residence (ref = Rural)						
Urban	0.010**	0.463***	0.041**	0.002	0.408***	0.002
	(0.004)	(0.464)	(0.020)	(0.008)	(0.006)	(0.010)
Subtotal			0.041			0.002
Women’s Education (ref =No education)						
Primary	−0.012	−0.033	−0.001	0.001	− 0.165***	0.000
	(0.010)	(0.025)	(0.002)	(0.004)	(0.018)	(0.002)
Secondary	0.018	0.271***	0.015	0.021**	0.234***	0.017**
	(0.017)	(0.014)	(0.015)	(0.010)	(0.011)	(0.008)
Higher	0.002*	0.803***	0.005*	0.013**	0.739***	0.033**
	(0.001)	(0.040)	(0.003)	(0.006)	(0.024)	(0.015)
Subtotal			0.021			0.050
Wealth quintile (ref= poorest)						
Poorer	−0.017	− 0.026***	0.001	0.014	−0.025***	−0.001
	(0.021)	(0.009)	(0.002)	(0.013)	(0.006)	(0.001)
Middle	0.017	−0.323***	−0.017	0.003	−0.378	−0.003
	(0.011)	(0.014)	(0.011)	(0.004)	(0.011)	(0.005)
Richer	0.017	0.087***	0.005	0.010*	0.025*	0.001*
	(0.011)	(0.012)	(0.003)	(0.005)	(0.015)	(0.001)
Richest	0.045***	0.486***	0.068***	0.026***	0.421***	0.038***
	(0.011)	(0.012)	(0.017)	(0.006)	(0.012)	(0.009)
Subtotal			0.057			0.035
NHIS covered (ref= No)						
Yes	0.030***	0.847***	0.080***	0.042***	0.809***	0.119***
	(0.010)	(0.007)	(0.026)	(0.012)	(0.007)	(0.033)
Subtotal			0.080			0.119
Age group of women (ref =15-19)						
20–24	−0.008	− 0.038	0.001	0.003	−0.083***	− 0.001
	(0.018)	(0.025)	(0.003)	(0.007)	(0.021)	(0.002)
25–29	0.017	0.046***	0.003	0.012	0.047***	0.002
	(0.021)	(0.021)	(0.004)	(0.011)	(0.017)	(0.002)
30–34	0.124	0.012***	0.005	0.009	0.092***	0.003
	(0.017)	(0.031)	(0.007)	(0.010)	(0.017)	(0.004)
35–39	0.014	−0.013	−0.001	0.009	0.048**	0.002
	(0.015)	(0.030)	(0.002)	(0.008)	(0.020)	(0.002)
40–44	0.009	−0.146***	− 0.004	0.007	−0.109***	− 0.003
	(0.008)	(0.043)	(0.003)	(0.004)	(0.036)	(0.002)
45–49	0.001	−0.312***	−0.001	0.002	−0.332***	− 0.002
	(0.004)	(0.066)	(0.004)	(0.002)	(0.060)	(0.001)
Subtotal			0.003			0.001
Region of residence (ref= western)						
Central	0.006	0.094***	0.002	0.004	0.061***	0.001
	(0.006)	(0.028)	(0.002)	(0.004)	(0.021)	(0.001)
Greater Accra	0.011*	0.650***	0.023*	−0.019***	0.561***	− 0.038***
	(0.006)	(0.018)	(0.012)	(0.006)	(0.014)	(0.011)
Volta	0.005	−0.149***	−0.002	−0.008***	− 0.197***	0.006***
	(0.005)	(0.029)	(0.003)	(0.002)	(0.018)	(0.002)
Eastern	−0.001	0.045*	0.000	−0.011***	−0.049***	0.002**
	(0.006)	(0.023)	(0.001)	(0.002)	(0.014)	(0.001)
Ashanti	0.012	0.146***	0.011**	0.000	0.254***	0.000
	(0.009)	(0.019)	(0.005)	(0.006)	(0.016)	(0.005)
Brong Ahafo	0.007	−0.107***	0.006	0.001	−0.192***	0.000
	(0.007)	(0.033)	(0.004)	(0.003)	(0.018)	(0.002)
Northern	0.013	−0.468***	−0.002	−0.007*	−0.643***	0.014*
	(0.008)	(0.024)	(0.002)	(0.003)	(0.010)	(0.008)
Upper East	0.011***	−0.533***	−0.018	0.006***	−0.666***	−0.014***
	(0.004)	(0.036)	(0.013)	(0.002)	(0.014)	(0.004)
Upper West	0.008***	−0.407***	−0.019***	0.001	−0.528***	−0.002
	(0.002)	(0.028)	(0.007)	(0.001)	(0.018)	(0.002)
Subtotal			0.001			−0.031

Robust standard errors in parenthesis. *** *p* < 0.01, **p < 0.05, *p < 0.10

**Table 4:** Contributing factors of inequality in DTA in Ghana, between 2008 and 2014

Variables	2008			2014		
	Elasticity	Concentration Index	Absolute contribution	Elasticity	Concentration Index	Absolute contribution
Place of residence (ref = Rural)						
Urban	0.080	0.464***	0.100	0.002	0.408***	0.002
				(0.009)	(0.006)	(0.011)
Subtotal			0.100			0.002
Women’s Education (ref = No education)						
Primary	0.015	−0.033	−0.001	0.001	−0.165***	0.000
	(0.012)	(0.025)	(0.002)	(0.005)	(0.018)	(0.002)
Secondary	0.032	0.271***	0.024	0.024**	0.234***	0.017**
	(0.020)	(0.014)	(0.015)	(0.012)	(0.011)	(0.008)
Higher	0.003**	0.803***	0.006**	0.015**	0.739***	0.033**
	(0.001)	(0.040)	(0.003)	(0.007)	(0.024)	(0.015)
Subtotal			0.029			0.050
Wealth quintile (ref= poorest)						
Poorer	0.004	−0.026***	0.000	0.016	−0.025***	−0.001
	(0.022)	(0.009)	(0.002)	(0.016)	(0.006)	(0.001)
Middle	0.076***	−0.323***	− 0.067***	0.003	−0.378***	−0.003
	(0.013)	(0.014)	(0.012)	(0.004)	(0.011)	(0.005)
Richer	0.096***	0.087***	0.023***	0.011*	0.025*	0.001
	(0.015)	(0.012)	(0.005)	(0.006)	(0.015)	(0.001)
Richest	0.120***	0.486***	0.158***	0.031***	0.421****	0.038***
	(0.016)	(0.012)	(0.022)	(0.007)	(0.013)	(0.009)
Subtotal			0.114			0.035
NHIS covered (ref = No)						
Yes	0.086***	0.847***	0.198***	0.050***	0.809***	0.119***
	(0.012)	(0.007)	(0.028)	(0.014)	(0.007)	(0.033)
Subtotal			0.198			0.119
Age group of women (ref =15-19)						
20–24	−0.018	− 0.038	0.002	0.004	−0.083***	− 0.001
	(0.018)	(0.025)	(0.002)	(0.008)	(0.021)	(0.002)
25–29	−0.024	0.046**	−0.003	0.014	0.047***	0.002
	(0.024)	(0.022)	(0.004)	(0.013)	(0.017)	(0.002)
30–34	−0.006	0.124***	−0.002	0.010	0.092***	0.003
	(0.019)	(0.031)	(0.006)	(0.013)	(0.017)	(0.004)
35–39	−0.003	−0.013	0.000	0.011	0.048**	0.002
	(0.014)	(0.031)	(0.002)	(0.010)	(0.020)	(0.002)
40–44	−0.002	−0.146***	0.001	0.008	−0.109***	− 0.003
	(0.008)	(0.043)	(0.003)	(0.005)	(0.036)	(0.002)
45–49	−0.006	− 0.312***	0.005	0.002	−0.332***	−0.002
	(0.005)	(0.066)	(0.004)	(0.002)	(0.060)	(0.002)
Subtotal			0.003			0.001
Region of residence (ref= western)						
Central	−0.001	0.094***	0.000	0.004	0.061***	0.001
	(0.008)	(0.028)	(0.002)	(0.004)	(0.021)	(0.001)
Greater Accra	0.005	0.650***	0.008	−0.023***	0.561***	−0.038***
	(0.007)	(0.018)	(0.012)	(0.007)	(0.014)	(0.011)
Volta	0.001	−0.149***	− 0.004	− 0.010***	− 0.197***	0.006***
	(0.007)	(0.029)	(0.003)	(0.003)	(0.018)	(0.002)
Eastern	0.004	0.045*	0.001	−0.013***	−0.049***	0.002**
	(0.008)	(0.023)	(0.001)	(0.003)	(0.014)	(0.001)
Ashanti	0.010	0.146***	0.013	0.000	0.254***	0.000
	(0.012)	(0.019)	(0.007)	(0.007)	(0.016)	(0.005)
Brong Ahafo	0.003	−0.107***	0.004	0.001	−0.192***	0.000
	(0.008)	(0.033)	(0.005)	(0.003)	(0.018)	(0.002)
Northern	−0.013	− 0.468***	− 0.001	−0.008*	− 0.643***	0.014*
	(0.012)	(0.024)	(0.003)	(0.004)	(0.010)	(0.008)
Upper East	−0.003	−0.533***	0.017	0.007***	−0.666***	−0.014***
	(0.004)	(0.036)	(0.016)	(0.002)	(0.014)	(0.004)
Upper West	0.004	−0.407***	0.004	0.001	−0.528***	−0.002
	(0.002)	(0.029)	(0.006)	(0.001)	(0.018)	(0.002)
Subtotal			0.042			−0.031

Robust standard errors are in parenthesis. *** *p* < 0.01, **p < 0.05, *p < 0.10

We found that NHIS coverage was among the largest contributors to socioeconomic inequality in ANC visit in Ghana. The results in Table 3 show a strong positive relationship between health insurance and required ANC visit (at least 4 visits). The elasticity of the NHIS variable was positive and significant at 1% in both 2008 and 2014.There was also evidence of significant inequality in NHIS membership. The CI for NHIS was 0.85 in 2008 and 0.81 in 2014. The positive CIs suggest that the inequality in NHIS coverage was in favor of the privileged. In absolute terms, NHIS contributed about 0.08 to inequality in required ANC visit in 2008. The contribution of NHIS to inequality in ANC utilization was higher in 2014 (0.12). These were both significant at the 1% level.

Other important contributors to socioeconomic related inequality in ANC utilization were education, wealth status, and urban location. For instance, the estimates suggest that wealth status alone contributed about 0.06 to inequality while urban location and education together contributed about 0.06 in 2008. In 2014, the contribution changed with education being the largest contributor. Education alone in 2014 contributed about 0.05 to inequality while urban location and wealth status contributed about 0.06. The CIs of these variables also suggest that, in general, there was significant concentration among the privileged. A graphical presentation of the absolute contributions of each of the covariates is reported in the appendix.

In Table 4, we present decomposition results for delivery by trained birth attendants. Similar to the case of ANC, results are presented for both 2008 and 2014. For each year the elasticities, CIs and absolute contributions are reported. The results show that NHIS coverage is one of the key contributors to inequality in DTA. The elasticity of the NHIS variable was positive and strongly significant in both 2008 and 2014. This suggests that women who were enrolled on the NHIS were more likely to seek skilled delivery services. The estimated elasticities for 2008 and 2014 were 0.09 and 0.05, respectively. Furthermore, the results indicate that NHIS also contributes positively to inequality in DTA service utilization. The absolute contribution from NHIS to this inequality was about 0.20 and 0.12 in 2008 and 2014, respectively. These estimates were also statistically significant at 1%.

Again, we found household wealth status, urban location and education to be significant in explaining inequality in DTA service utilization. Wealth status contributed about 0.11 to inequality in 2008 and 0.04 in 2014. Urban residence contributed about 0.10 to inequality in 2008 and 0.002 in 2014, even though these were not statistically significant. In terms of education only attainment of higher than secondary education was significant contributor to inequality. However, in 2014, both secondary education and higher education were significant contributors. The statistics suggest that, in absolute terms, education contributed about 0.03 and 0.05, respectively, to inequality in DTA utilization.

## Discussion

The study set out to measure and explain socioeconomic inequalities in maternal healthcare service use in Ghana. The findings showed that there exist wealth-related inequalities in at least four ANC visit and DTA. The estimates suggest that these inequalities were pro-rich implying women from richer households were advantaged in the use of these two services compared to their poor counterparts. However, pro-rich inequalities in both at least four ANC visits and DTA decreased in 2014 compared to the levels in 2003.

The results corroborate previous studies that show pro-rich inequalities in maternal health care utilization in Ghana [8]. More importantly, it also underscores global goals that seek to leave no one behind. As indicated earlier, the SDGs outline goals and targets that will help mitigate these inequalities. The findings of this study provide important evidence to highlight the nature of these inequalities and identify determinants. The decomposition results also provide significant emphasis on the contribution of the NHIS in addressing socioeconomic inequality. Among all the variables used in the decomposition, access to NHIS contributed the largest to inequality in ANC visits and DTA. This suggests that reducing financial barriers to use ANC and DTA services reduces the inequality gap. This may be explained by policy efforts in 2008 that sought to completely remove financial barriers for pregnant women registered under the NHIS. This enabled women from rural and less privileged backgrounds to seek care. Existing evidence suggests that the NHIS has significant impact on maternal health care utilization [22]. Before 2008, both pre and post-natal women, irrespective of the economic background were required to pay premiums to enroll on the NHIS or pay out of pocket to access health care. This created a major barrier for poor and vulnerable women who end up seeking alternative care from less skilled traditional sources where the risk of complications and eventual mortality was high. There was also the risk of catastrophic spending among women who attempted to pay out of pocket for ANC and post-natal services. The initiative in 2008 was therefore relevant and crucial for improving maternal health in Ghana.

While our findings point to the fact that sustaining and scaling up the NHIS would reduce the inequality gap, there is need to ensure effective operations of the scheme. For instance, while pregnant women may be officially exempted from paying premiums, there may be unofficial payments that discourages service utilization. Furthermore, the findings highlight the need to look beyond financial barriers to infrastructure barriers. The fact that inequality still persists in the presence of free pregnancy related services may be partly due to lack of adequate health facilities. In some deprived rural communities, women face the challenge of walking long distances to access health care, even though services are free. Ensuring that health facilities are provided within reasonable distances will be a step in the right direction.

A starting point will be resourcing and extending the Community-based Health Planning and Services (CHPS) programme. The CHPS was designed to provide basic health care to deprived communities that otherwise did not have easy access to health facilities [13]. It was designed to service as the first line of care with capacity to provide ANC services and uncomplicated vaginal delivery. This has proved to be an important intervention to provide primary health care, especially in deprived rural communities (Johnson et al. [11]. Unfortunately, there remain major challenges with the implementation of the intervention with facilities lacking basic infrastructure and skilled workforce. Addressing these limitations and ensuring the programme is rolled out across the country will be a significant step towards bridging the inequality gap.

The study also showed that education was the other important factor that account for the inequalities in ANC visits and DTA. This is not surprising as a mother’s decision to seek care during pregnancy depends, to some extent, on the level of education. Several studies have identified education as a major determinant of maternal health and health seeking behavior [6, 10, 16, 19]. Unfortunately, in developing countries like Ghana, women from poorer household also have lower educational attainments relative to their counterparts from rich households. This suggests that to improve inequalities in health care, there is need to also ensure that educational policies target women from poor and deprived households.

Current efforts in this direction worth noting is the recently rolled out free secondary education programme in Ghana. This intervention is targeted at children who would have dropped out of school for financial reasons. The government provides all financial costs related to secondary education and this has significantly increased secondary school enrolment in the country. This has also come as a major relief for parents who could not afford school fees at the secondary. If well implemented, the intervention will likely reduce inequality in education and, ultimately, reduce inequality in accessing maternal health care.

## Conclusion

This study examined socioeconomic inequalities in maternal health care service utilization in Ghana. We sought to identify the key factors that account for these inequalities. We found evidence of pro-rich inequality in ANC utilization and DTA but trend analysis reveal that it decreased in 2014. The decomposition analysis showed that NHIS coverage and education were the major contributors to inequality in ANC utilization and DTA. This suggests that removing financial barriers through the NHIS is relevant for bridging the gap in service utilization. This also indicates that improving the performance and coverage of the NHIS will be a good step towards achieving universal health coverage in Ghana. Moreover, ensuring that policies that improve education target the poor will be relevant in reducing inequalities in maternal health care utilization.

## Figures and Tables

**Fig. 2:**
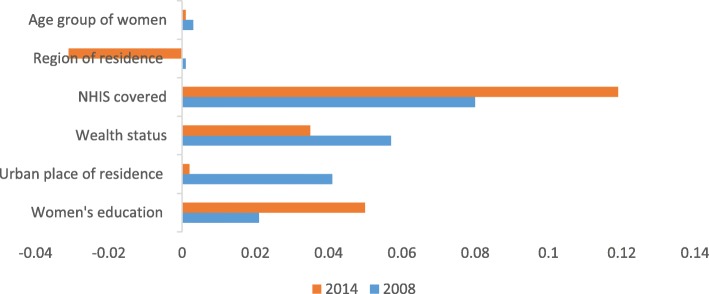
Absolute contribution to socio-economic related inequalities in required ANC visit

**Fig. 3:**
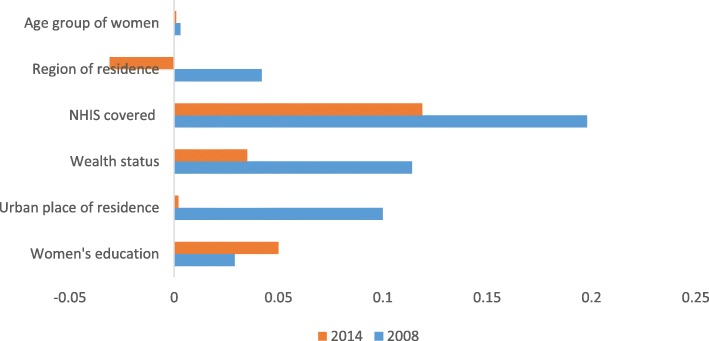
Absolute contribution to socio-economic related inequalities in DTA service utilization

## Data Availability

The datasets analysed during the current study are available in the DHS Program repository, https://dhsprogram.com/data/.

## Abbreviations

ANC: Antenatal Care
CC: Concentration Curve
CI: Concentration Index
DHS: Demographic and Health Survey
DTA: Delivery by Trained Attendants
EA: Enumeration Area
GDHS: Ghana Demographic and Health Survey
GHS: Ghana Health Service
NHIS: National Health Insurance Scheme
NPHRL: National Public Health and Reference Laboratory
UHC: Universal Health Coverage
WHO: World Health Organization

## Footnotes

1 We have omitted the 2003 estimates as data was collected before the introduction of NHIS (one of the key variables of interest).
